# Genomic insights into the global dissemination of linezolid resistance genes in *Enterococcus faecium*

**DOI:** 10.3389/fmicb.2026.1783172

**Published:** 2026-04-07

**Authors:** Xiaoxiao Yuan, Chao Ma, Xiaoli Cao, Zhiwei Ma, Liuli Wu, Peitao Jiang, Zhengnan Yang

**Affiliations:** 1 Department of Laboratory, Nanjing Drum Tower Hospital Group, Yizheng Hospital, Yangzhou, China; 2 Nanjing University Medical School Affiliated Nanjing Drum Tower Hospital, Nanjing, China

**Keywords:** CFR, *Enterococcus faecium*, genomic epidemiology, horizontal gene transfer, linezolid resistance, mobile genetic elements, *optrA*, *poxtA*

## Abstract

**Background:**

Transferable linezolid resistance determinants in *Enterococcus faecium*, particularly *optrA, poxtA*, and *cfr*, have raised increasing global concern due to their potential dissemination across clinical, animal, food, and environmental reservoirs. However, their population distribution, genomic contexts, and associated virulence backgrounds remain incompletely understood.

**Methods:**

We performed a large-scale genomic analysis of 2,235 publicly available *E. faecium* genomes to investigate the distribution, sequence types (STs), virulence factors (VFs), and genomic contexts of transferable linezolid resistance genes. Phylogenetic analysis and comparative examination of gene flanking regions were conducted to explore the mechanisms underlying resistance gene dissemination.

**Results:**

A total of 243 genomes (10.9%) carried at least one linezolid resistance gene. poxtA (8.1%) and *optrA* (6.9%) were more frequently detected than *cfr* (2.2%). Multiple resistance determinants were simultaneously observed within individual isolates, with *optrA* + *poxtA* representing the most frequent gene combination. These resistance genes were distributed across 90 distinct STs, indicating substantial population diversity. Comparative phylogenetic and genomic context analyses revealed significant genetic diversity and a lack of strict phylogenetic clustering, suggesting that horizontal gene transfer mediated by mobile genetic elements plays a major role in the dissemination of these determinants. VF analysis identified 44 virulence genes, with several adherence-associated genes highly conserved across isolates. Notably, certain adherence-related VFs were enriched in isolates harboring multiple resistance genes.

**Conclusion:**

Together, these findings highlight the widespread dissemination of *optrA*-, *poxtA*-, and *cfr*-mediated linezolid resistance in diverse *E. faecium* genomic backgrounds and underscore the important role of horizontal gene transfer in their spread. Continuous genomic surveillance integrating resistance and virulence analyses will be essential for understanding and controlling the global transmission of linezolid-resistant *E. faecium*.

## 1 Introduction

*Enterococcus faecium* is a major opportunistic pathogen responsible for a wide range of nosocomial infections, including bloodstream infections, urinary tract infections, and intra-abdominal infections (Wei et al., 2024). Owing to its intrinsic resistance to multiple antimicrobial agents and its remarkable capacity to acquire additional resistance determinants, *E. faecium* has emerged as a significant clinical challenge worldwide (Freitas et al., 2021). Linezolid, an oxazolidinone antibiotic, is considered one of the last-resort therapeutic options for the treatment of infections caused by multidrug-resistant Gram-positive bacteria (Hashemian et al., 2018), including vancomycin-resistant *enterococci* (VER). However, the increasing emergence of linezolid-resistant *E. faecium* threatens its clinical utility and raises serious public health concerns.

Linezolid resistance in *E. faecium* is mediated by both chromosomal mutations and transferable resistance genes (Lázaro-Perona et al., 2025). Among these, the plasmid- or transposon-associated genes *optrA*, *poxtA*, and *cfr* are of particular concern because of their ability to confer resistance to linezolid and other clinically important antimicrobials and to disseminate via horizontal gene transfer (Coccitto et al., 2023). The *optrA* gene, first reported in 2015, encodes an ATP-binding cassette F protein that protects the ribosome from oxazolidinones and phenicols (Wang et al., 2015). Similarly, *poxtA* confers resistance to oxazolidinones, phenicols, and tetracyclines (Shan et al., 2024), while *cfr* encodes a methyltransferase that modifies the 23S rRNA and mediates resistance to multiple classes of antibiotics (Kaminska et al., 2010). The rapid global spread of these genes in clinical, animal, food, and environmental isolates highlight their epidemiological importance and underscores the importance of understanding their dissemination mechanisms.

Previous studies investigation of linezolid resistance genes in *E. faecium* have largely focused on localized outbreaks, specific geographic regions, or limited collections of isolates (Egan et al., 2020; Freitas et al., 2020; Mortelé et al., 2024; Beh et al., 2025). Although these studies have provided valuable insights, comprehensive large-scale analyses integrating global genomic datasets, population structure, and comparative gene-context analysis remain limited. In particular, the evolutionary relationships of *optrA*-, *poxtA*-, and *cfr-*positive *E. faecium* strains and the relative contributions of clonal expansion versus horizontal gene transfer to the dissemination of these resistance genes have not been systematically evaluated at a global scale. Moreover, it remains unclear whether linezolid resistance gene–carrying isolates differ from resistance gene–negative isolates in their virulence gene profiles, which may have implications for their pathogenic potential and clinical impact.

To address these questions, we conducted a large-scale genomic analysis of 2,235 high-quality *E. faecium* genomes collected from multiple sources worldwide to investigate the distribution, STs, epidemiological characteristics, and evolutionary dynamics of the linezolid resistance genes *optrA*, *poxtA*, and *cfr*. By integrating core genome SNP-based phylogenetic analysis, sequence typing, metadata analysis, comparative analysis of resistance gene flanking regions, and virulence factor (VF) profiling, we aimed to characterize the genetic diversity and population structure of resistance gene–carrying strains.

Specifically, this study addressed the following research questions: (i) how are the transferable linezolid resistance genes *optrA, poxtA*, and *cfr* distributed across the global *E. faecium* population and different sequence types (STs); (ii) what are the evolutionary relationships among resistance gene–carrying strains; (iii) to what extent does horizontal gene transfer mediated by mobile genetic elements contribute to the global dissemination of these resistance determinants compared with clonal expansion; and (iv) whether *optrA*-, *poxtA*-, and *cfr*-positive *E. faecium* isolates differ from resistance gene–negative isolates in their VF carriage patterns. Addressing these questions will provide new insights into the evolutionary dynamics, dissemination mechanisms, and potential pathogenic characteristics of linezolid-resistant *E. faecium*.

## 2 Materials and methods

### 2.1 Genome sequence download and processing

All *E. faecium* genome sequences available in GenBank format were downloaded in batch from the NCBI genome database^1^ using Aspera (Sayers et al., 2025), with a cutoff date of December 7, 2023. A total of 3,256 genome sequences were collected. A Perl script was used to extract nucleotide sequence files from all GenBank files (Stajich et al., 2002). To ensure consistency in gene prediction and avoid biases introduced by heterogeneous annotation pipelines, all genomes were re-annotated using Prodigal v2.6.3 (Hyatt et al., 2010) with default parameters. Genome quality was assessed using CheckM v1.1.3 and QUAST v5.0.2 (Gurevich et al., 2013; Parks et al., 2015). High-quality genomes were defined as having completeness > 90% and contamination < 5%, which are commonly used thresholds for bacterial genome quality assessment (Bowers et al., 2017). In addition, genomes were required to contain ≤ 500 contigs and an N50 value ≥ 40 kb to ensure sufficient assembly continuity for downstream comparative analyses (Gurevich et al., 2013). After quality filtering, 2,235 high-quality genomes were retained for subsequent analyses (Supplementary Table S1).

### 2.2 Antimicrobial resistance gene analysis

All antimicrobial resistance gene sequences were downloaded from the NCBI pathogen antimicrobial resistance gene database and compiled into a structured local AMR database. Resistance gene detection was performed using BLASTN (Camacho et al., 2009). Nucleotide coding sequences predicted by Prodigal annotation from each of the 2,235 genomes were aligned against the antimicrobial resistance database to identify the distribution of *cfr*, *poxtA*, and *optrA* genes across all genomes (Hyatt et al., 2010). Hits were considered valid if they met all of the following criteria: *E*-value ≤ 1e-5, nucleotide identity ≥ 90%, alignment coverage ≥ 90% of the reference gene length, and minimum alignment length ≥ 30 bp (Altschul et al., 1997). The minimum match length threshold was used to ensure the detection of potential gene fragments while avoiding spurious short matches. However, only hits meeting the identity and coverage thresholds were retained for downstream analyses, and partial or truncated gene fragments that did not satisfy the ≥ 90% coverage criterion were excluded.

To assess co-occurrence patterns of transferable linezolid resistance genes, the presence of *optrA*, *poxtA*, and *cfr* within individual genomes was examined. Isolates were categorized based on single-gene carriage or multi-gene combinations, and frequencies were calculated among gene-positive genomes.

### 2.3 Sequence typing analysis

Multilocus sequence typing (MLST) was performed on all 2,235 genomes by the *E. faecium* MLST scheme available in the pubMLST database (Achtman scheme, accessed on December 10, 2023). The sequences of the seven housekeeping genes (*adk, atpA, ddl, gdh, gyd, purK, and pstS*) and the corresponding allele profile file were downloaded from pubMLST *Enterococcus faecium* MLST scheme.^2^ The ST_tool identifies allele matches using BLASTN, requiring 100% sequence identity and 100% coverage for allele assignment (Camacho et al., 2009). The detected alleles were then matched to the pubMLST allele profile database to determine the corresponding STs. To validate the accuracy of the ST_tool, sequence typing results were cross-checked against the pubMLST typing results for a subset of genomes (*n* = 100),^3^ showing consistent ST assignments. The tool has been designed to automate allele detection and ST assignment for large genome datasets.

### 2.4 Virulence gene analysis

VFs were identified using whole-genome sequencing data. The assembled genome sequences of all isolates were screened against the Virulence Factor Database (VFDB) using ABRicate^4^ with default parameters. A gene was considered present when the sequence showed a minimum identity of ≥ 90% and coverage of ≥ 80% relative to the reference sequence. The detected VFs were further categorized into six functional groups based on their known biological roles, including adherence, biofilm formation, exoenzyme production, exotoxin production, immune modulation, and stress survival. The presence of VFs in each isolate was subsequently summarized in a Microsoft Excel spreadsheet, and the number and percentage of isolates carrying each VF were calculated. The prevalence of VFs was determined for the overall isolate collection as well as for different groups of isolates. To investigate the relationship between VFs and linezolid resistance determinants, isolates were classified into four groups: linezolid resistance gene–negative isolates and isolates carrying one, two, or three linezolid resistance genes. The prevalence of each VF among these groups was compared. Differences in VF prevalence between groups were analyzed using the chi-square test or Fisher’s exact test, as appropriate. A *p* < 0.05 was considered statistically significant.

### 2.5 Extraction and processing of metadata

Associated metadata were extracted from GenBank files of the 2,235 genomes using a custom Perl script. In total, 243 isolates carrying *cfr*, *poxtA*, or *optrA* were identified. Metadata including isolation year, country of origin, host, sample source, and ST were compiled into a single table for further analysis. For genomes with incomplete metadata (e.g., missing host or country information), missing values were recorded as “unknown.” Such genomes were included in all analyses except those requiring the corresponding metadata fields.

### 2.6 Phylogenetic and comparative genomic analysis

Orthologous gene analysis was conducted using OrthoFinder v2.5.5 (Emms and Kelly, 2019), resulting in the identification of single-copy core genes shared among the analyzed genomes. Core gene alignments were concatenated, and single nucleotide polymorphisms (SNPs) were extracted using SNP-sites v2.5.1 (Page et al., 2016). A maximum-likelihood phylogenetic tree was constructed using RAxML-NG v1.2.1 (Kozlov et al., 2019) under the GTR + G substitution model with 1,000 bootstrap replicates to assess branch support. For visualization of resistance gene genetic environments, genomic regions flanking *cfr*, *poxtA*, or *optrA* (± 10 kb) were extracted and analyzed using Gcluster (Li et al., 2020), together with the phylogenetic tree and associated metadata. Representative isolates were selected to reduce redundancy based on clustering of highly similar genomic contexts (≥ 99% nucleotide identity across the extracted region). This selection strategy may reduce overrepresentation of nearly identical isolates but could potentially underestimate within-cluster diversity.

## 3 Results

### 3.1 Distribution of linezolid resistance genes in *E. faecium*

Among the 2,235 publicly available *E. faecium* genomes analyzed in this study, 243 isolates (10.9%) harbored at least one transferable linezolid resistance gene. Specifically, *poxtA* was detected in 182 genomes (8.1%), *optrA* in 155 genomes (6.9%), and *cfr* in 50 genomes (2.2%). These proportions reflect frequencies within the analyzed genomic dataset and should not be interpreted as population-level prevalence estimates.

In addition, several isolates carried multiple linezolid resistance genes simultaneously. Among the 243 gene-positive isolates, 126 isolates carried a single resistance gene, whereas 91 isolates harbored two genes and 26 isolates carried all three genes. The most common gene combination was *optrA* + *poxtA*, observed in 72 isolates, followed by *optrA* + *cfr* and *poxtA* + *cfr*, observed in 13 and 6 isolates, respectively. It should be noted that these frequencies reflect the distribution among publicly available genomes included in this dataset and do not necessarily represent the true global prevalence of these resistance genes in *E. faecium* populations.

### 3.2 Sequence typing of linezolid resistance gene–carrying *E. faecium*

The 243 gene-positive isolates were assigned to 90 distinct STs, demonstrating substantial population diversity. ST22 was the most frequently detected ST (12/244, 4.9%), followed by ST324 (8/244, 3.3%), ST104 (7/244, 2.9%), and ST156 and ST1991 (6/244 each, 2.5%).

The 50 *cfr*-positive isolates were distributed among five STs, with ST203 being the most common (3/50, 6.0%). The 182 *poxtA*-positive isolates belonged to 67 STs, predominantly ST22 (11/182, 6.0%). Similarly, the 155 *optrA*-positive isolates were classified into 59 STs, with ST22 being the most frequently detected (9/155, 5.8%) (Table 1).

**TABLE 1:** Distribution of *cfr*, *poxtA*, and *optrA* among predominant sequence types of *Enterococcus faecium*.

Gene	Sequence type (ST)	No. of isolates (n)	Percentage (%)
*cfr*	ST203	3	0.13
	ST2329	2	0.09
	ST17	1	0.04
	ST32	1	0.04
	ST1846	1	0.04
*poxtA*	ST22	11	0.49
	ST324	7	0.31
	ST104	7	0.31
	ST156	6	0.27
	ST1991	6	0.27
*optrA*	ST22	9	0.40
	ST324	6	0.27
	ST1991	6	0.27
	ST104	5	0.22
	ST650	3	0.13

### 3.3 Distribution of virulence genes among isolates

A total of 44 VFs belonging to six functional categories were detected among the isolates, including adherence (28 genes), biofilm formation (1 gene), exoenzyme (1 gene), exotoxin (6 genes), immune modulation (7 genes), and stress survival (1 gene)

Several VFs were highly prevalent in both linezolid resistance gene–negative and linezolid resistance gene–positive isolates. In particular, *eno*, *plr/gapA*, and efaA (adherence-associated genes), *cpsB/cdsA* (immune modulation–related gene), and bopD (biofilm-associated gene) were detected in nearly all isolates, with prevalence rates exceeding 98% in both groups, indicating that these genes represent conserved virulence determinants in the studied population. In contrast, a group of adherence-related genes showed moderate prevalence, including *acm*, *pilA*, and *scm*, with detection rates ranging from approximately 19 to 80% among isolates. Several virulence genes were rarely detected, including *asa1*, *esp*, bee1, and *ebpB* (adherence-related genes), *eno* (exoenzyme-related gene), *ace*, *cylA*, *cylB*, *cyll, cylM*, and *cylR2* (exotoxin-associated genes), and *BCE_RS25860, SH_RS01820, and STER_RS05250* and *rfbB* (immune modulation–related gene), which were present in fewer than 4.0% of isolates. Notably, all exotoxin-associated genes were absent in the majority of isolates.

To further explore the relationship between antimicrobial resistance and VFs, the distribution of VFs was compared among isolates carrying different numbers of linezolid resistance genes (one, two, or three genes). Several VFs showed significant differences in prevalence between linezolid resistance gene–negative isolates and isolates carrying resistance genes (Table 2). Among the adherence-associated genes, significant differences were observed for *ACI49664*, *ACI49667*, *ACI49672*, *EFME1162_RS23495*, *acm*, *Bee2*, *Bee3*, *ecbA/fss3*, *sgrA*, *Srt1*, *Srt2*, *tuf*, and *tufA*. Notably, the prevalence of *ecbA/fss3* and *sgrA* increased markedly in isolates harboring multiple resistance genes. For example, the prevalence of *ecbA/fss3* increased from 45.0% in linezolid resistance gene–negative isolates to 84.6% in isolates carrying three resistance genes (*p* < 0.0001). Similarly, *sgrA* increased from 55.1 to 92.3% in isolates carrying three resistance genes (*p* < 0.0001). In contrast, *bee2*, *bee3*, *srt1*, and *srt2* were more frequently detected in isolates carrying one or two resistance genes but were absent in isolates carrying three resistance genes.

**TABLE 2:** Distribution of virulence genes among *Enterococcus faecium* isolates according to linezolid resistance gene profiles.

Category	Virulence gene	Negative isolate n (%)	Linezolid resistance gene positive isolates			*P*-value		
			One gene n (%)	Two genes n (%)	Three genes n (%)	neg vs. one	neg vs. two	neg vs. three
Adherence	*ACI49664*	1,476 (74.1)	85 (67.5)	59 (64.8)	25 (96.2)	0.052^a^	0.028^a^*	0.011^a^*
Adherence	*ACI49667*	1,297 (65.1)	69 (54.8)	53 (58.2)	25 (96.2)	0.011^a^*	0.120^a^	0.003^a^*
Adherence	*ACI49668*	1,116 (56.0)	78 (61.9)	59 (64.8)	3 (11.5)	0.270^a^	0.130^a^	< 0.0001^a^*
Adherence	*ACI49669*	1,101 (55.3)	81 (64.3)	59 (64.8)	3 (11.5)	0.071^a^	0.090^a^	< 0.0001^a^*
Adherence	*ACI49670*	1,130 (56.7)	79 (62.7)	59 (64.8)	3 (11.5)	0.260^a^	0.160^a^	< 0.0001^a^*
Adherence	*ACI49672*	1,449 (72.7)	88 (69.8)	57 (62.6)	8 (30.8)	0.330^a^	0.018^a^*	< 0.0001^a^*
Adherence	*ACI49673*	1,157 (58.1)	81 (64.3)	59 (64.8)	3 (11.5)	0.240^a^	0.270^a^	< 0.0001^a^*
Adherence	*EFME1162_RS23495*	155 (7.8)	3 (2.4)	1 (1.1)	0 (0)	0.014^a^*	0.006^a^*	0.600^a^
Adherence	*Acm*	1,302 (65.4)	63 (50.0)	57 (62.6)	26 (100.0)	0.0003^a^*	0.450^a^	0.001^a^*
Adherence	*bee2*	164 (8.2)	27 (21.4)	30 (33.0)	0 (0)	< 0.0001^a^*	<0.0001^a^*	0.120^a^
Adherence	*bee3*	161 (8.1)	27 (21.4)	30 (33.0)	0 (0)	< 0.0001^a^*	<0.0001^a^*	0.140^a^
Adherence	*ecbA/fss3*	896 (45.0)	24 (19.0)	21 (23.1)	22 (84.6)	< 0.0001^a^*	<0.0001^a^*	< 0.0001^a^*
Adherence	*pilA*	1,343 (67.4)	87 (69.0)	57 (62.6)	8 (30.8)	0.910^a^	0.230^a^	< 0.0001^a^*
Adherence	*sgrA*	1,098 (55.1)	33 (26.2)	30 (33.0)	24 (92.3)	< 0.0001^a^*	<0.0001^a^*	< 0.0001^a^*
Adherence	*srt1*	165 (8.3)	27 (21.4)	28 (30.8)	0 (0)	< 0.0001^a^*	<0.0001^a^*	0.220^a^
Adherence	*srt2*	161 (8.1)	27 (21.4)	28 (30.8)	0 (0)	< 0.0001^a^*	<0.0001^a^*	0.250^a^
Adherence	*Tuf*	1,756 (88.2)	123 (97.6)	89 (97.8)	26 (100)	0.003^a^*	0.012^a^*	0.170^a^
Adherence	*Tufa*	197 (9.9)	3 (2.4)	2 (2.2)	0 (0)	0.003^a^*	0.012^a^*	0.190^a^
Stress survival	*Bsh*	1,808 (90.8)	120 (95.2)	89 (97.8)	26 (100)	0.220^a^	0.048^a^*	0.300^a^

Data are presented as n (%). Percentages for virulence gene–negative isolates were calculated based on the total number of genomes analyzed (*n* = 1,992). Percentages for linezolid resistance gene–positive groups were calculated within each corresponding subgroup.

^a^*P*-values were calculated using the χ^2^-test or Fisher’s exact test, as appropriate.

* Statistically significant (*P* < 0.05). Gene names are presented in *italics* according to standard bacterial gene nomenclature.

Additionally, *tuf* showed a consistently high prevalence across all groups but was significantly more frequent in resistance gene–positive isolates (*p* < 0.05), whereas *tufA* was detected at a significantly lower frequency in these isolates. Beyond adherence-associated genes, the stress survival gene *bsh* also demonstrated a significant difference between groups, with a higher prevalence in isolates carrying two resistance genes compared with resistance gene–negative isolates (97.8% vs. 90.8%, *p* = 0.048) (Table 2).

### 3.4 Epidemiological characteristics of linezolid resistance gene–positive *E. faecium*

Linezolid resistance gene–positive isolates were primarily derived from human -associated samples (52/244, 21.3%), followed by pigs (38/244, 15.6%) and cattle (16/244, 6.6%). Among human-derived samples, the most common sources were feces/rectal swabs (27 isolates, 1.21%), followed by urine (6 isolates, 0.27%) and blood (5 isolates, 0.22%) (Table 3).

**TABLE 3:** Host sources, geographic distribution, and human sample types of linezolid resistance gene–positive *Enterococcus faecium*.

Host distribution			Geographic distribution			Sample types among human isolates		
Host	No. of isolates (n)	Percentage (%)	Country/Region	No. of isolates (n)	Percentage (%)	Sample type	No. of isolates (n)	Percentage (%)
Human	52	2.33	China	74	3.31	Feces/rectal swab	27	1.21
Pig	38	1.70	Belgium	43	1.92	Urine	6	0.27
Cattle	16	0.72	Pakistan	41	1.83	Blood	5	0.22
Fly	3	0.13	France	15	0.67	Digestive tract	4	0.18
Chicken	4	0.18	Réunion (France)	9	0.40	Bile	2	0.09
–	–	–	India	6	0.27	Pus	2	0.09

Geographically, gene-positive isolates were most frequently identified in China (74/244, 30.3%), Belgium (43/244, 17.6%), and Pakistan (41/244, 16.8%) within the available dataset (Table 3). Within China, Jiangsu Province accounted for the highest number of isolates (23 isolates, 1.0%), followed by Sichuan Province (10 isolates, 0.5%) and Henan Province (4 isolates, 0.2%).

**FIGURE 1:**
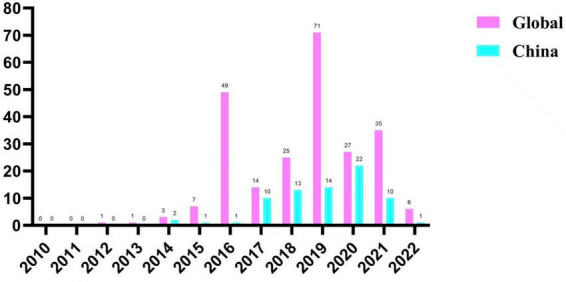
Temporal distribution of linezolid resistance gene–positive *Enterococcus faecium* isolates worldwide and in China, 2010 to 2022. The bar chart illustrates the yearly number of *Enterococcus faecium* isolates harboring linezolid resistance genes reported between 2010 and 2022. Pink bars indicate isolates reported worldwide, whereas cyan bars represent isolates reported in China. Numbers above the bars denote the total number of isolates identified each year.

Temporal analysis indicated increased detection of gene-positive genomes in publicly available databases around 2019 (71 isolates). However, these trends likely reflect sequencing activity and data submission patterns rather than true incidence. In China, the number of such isolates exhibited an overall increasing trend since 2014 and reached a peak in 2020, with 22 isolates (1.0%) (Figure 1).

**FIGURE 2:**
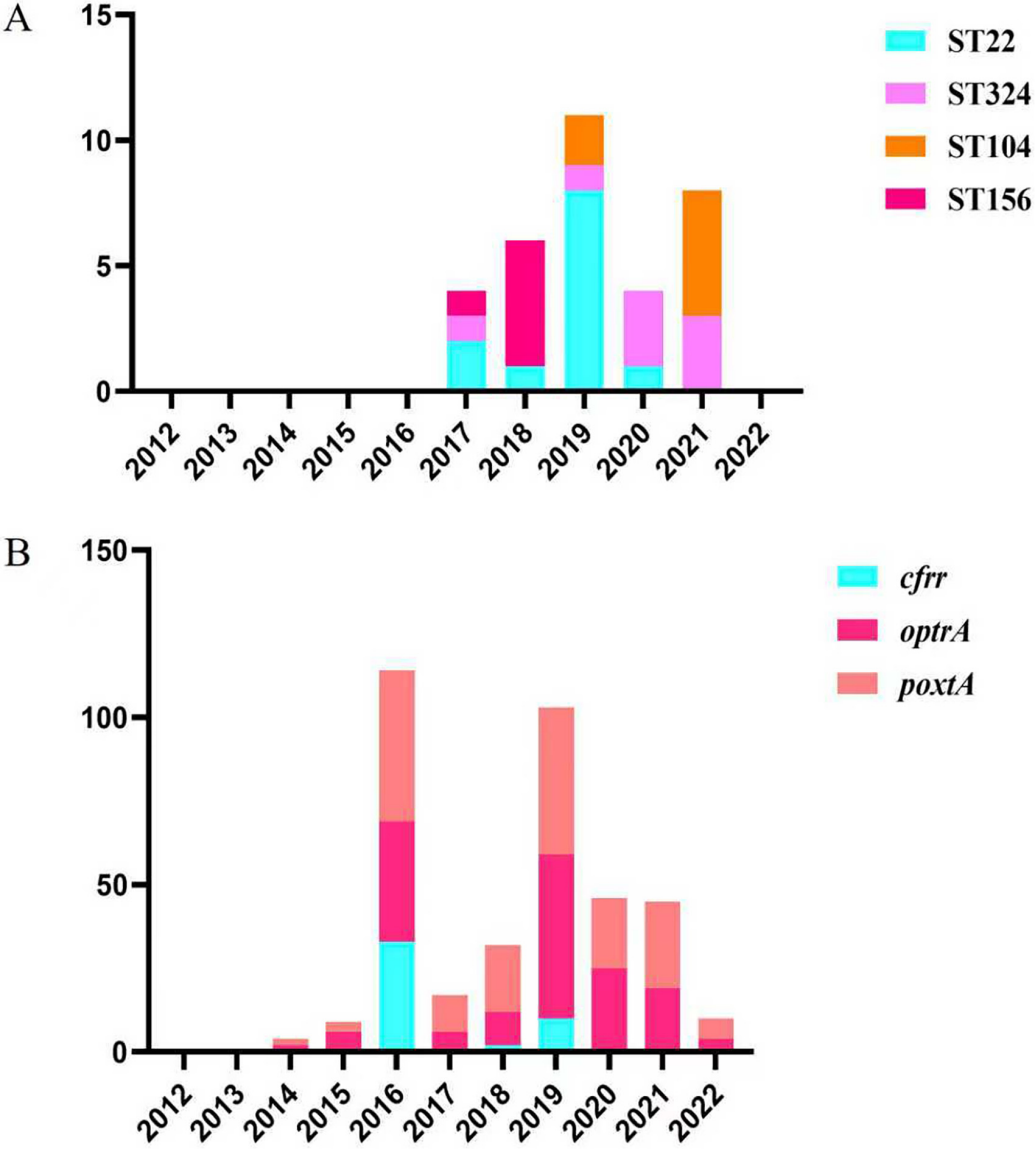
Temporal distribution of major linezolid resistance genes and predominant sequence types (STs), 2012 to 2022. **(A)** Annual distribution of predominant STs among linezolid-resistant *E. faecium* isolates during the same period. The stacked bars indicate the number of isolates belonging to major sequence types, including ST22, ST324, ST104, and ST156. **(B)** Annual distribution of major linezolid resistance genes identified in *Enterococcus faecium* isolates from 2012 to 2022. The stacked bars represent the number of isolates carrying different resistance genes, including *cfr*, *optrA*, and *poxtA*.

Regarding the emergence of individual resistance genes, *poxtA* was first detected in 2012, followed by *optrA* in 2013, whereas *cfr* appeared later in 2015. All three genes reached peak prevalence in 2016 and 2019 and have since persisted (Figure 2B).

Regarding ST dynamics, certain STs were more frequently observed during specific periods; however, the available genomic data do not allow definitive conclusions regarding epidemic expansion. Major epidemic clones emerged mainly during 2017–2018. ST156 peaked in 2018, ST22 was most prevalent in 2019, while ST324 and ST104 were most frequently detected during 2020–2021 (Figure 2A). ST156 predominantly carried *optrA* (3 isolates, 42.9%) and *poxtA* (6 isolates, 85.7%). ST22 mainly harbored *optrA* (9 isolates, 20.5%) and *poxtA* (11 isolates, 25.0%). ST324 was largely associated with *optrA* (6 isolates, 75.0%) and *poxtA* (7 isolates, 87.5%), whereas ST104 primarily carried *optrA* (5 isolates, 55.6%) and *poxtA* (7 isolates, 77.8%).

### 3.5 Phylogenetic diversity and conserved genomic context of *poxtA*-positive isolates

This study analyzed 182 *poxtA*-positive isolates, but due to incomplete sequencing, only 62 isolates with complete *poxtA* flanking sequences were selected for in-depth analysis. The resulting phylogenetic tree revealed significant genetic diversity, with isolates distributed across multiple clades, indicating that horizontal gene transfer likely plays a major role in *poxtA* dissemination, although a minor contribution of clonal expansion cannot be completely excluded (Supplementary Figure S1). Flanking region analysis showed both conserved and variable genetic structures among isolates, with some sharing similar sequences while others exhibited significant variation, including mobile genetic elements like insertion sequences and transposases.

**FIGURE 3:**
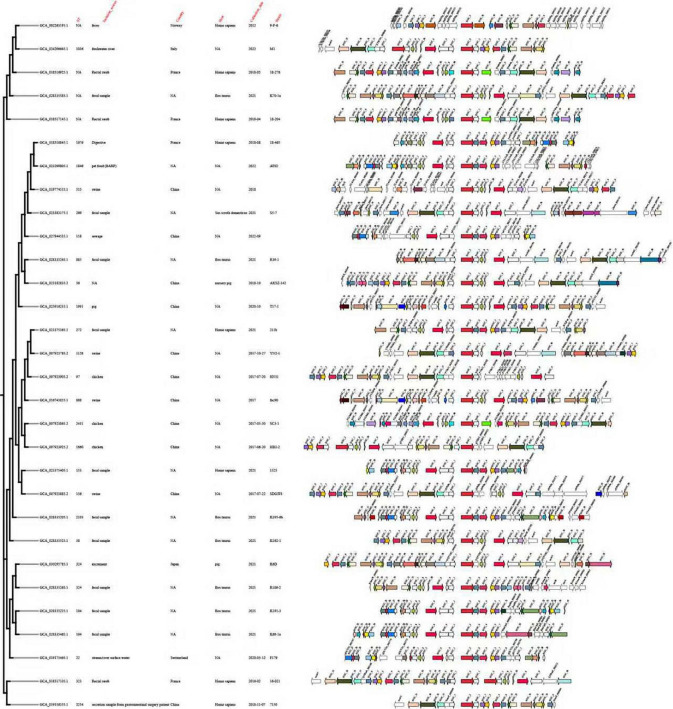
Phylogenetic relationships and flanking genetic environments of 30 *poxtA*-positive *Enterococcus faecium* isolates. A core genome single-nucleotide polymorphism (SNP)-based maximum-likelihood phylogenetic tree was constructed for 30 representative *poxtA*-positive *E. faecium* isolates selected based on distinct *poxtA*-associated genetic environments. Isolates that were genetically closely related and exhibited identical *poxtA* flanking structures were excluded to improve visualization clarity. The phylogenetic tree is shown on the left, with associated metadata including isolation source, country, host, collection year, and strain information. The genetic environments surrounding the *poxtA* gene are displayed on the right, with arrows representing predicted open reading frames and colored according to gene function. Despite the pronounced phylogenetic diversity among the isolates, the *poxtA*-associated genetic environments show a high degree of structural conservation, with *poxtA* frequently flanked by mobile genetic elements such as insertion sequences and transposase-related genes, although minor variations in gene content and organization are observed.

To avoid redundancy in visualization and comparative analysis, representative isolates were selected based on both phylogenetic diversity and variation in *poxtA*-flanking genetic structures. Isolates sharing nearly identical core genome phylogenetic positions and highly similar *poxtA*-associated genetic environments were considered redundant, and only one representative isolate was retained. Using this approach, 30 representative isolates were selected for detailed comparative analysis. The orthologous gene analysis of these 30 isolates showed that *poxtA* was widely disseminated across diverse bacterial populations, without clustering based on host origin, geographic location, or isolation year, suggesting that horizontal gene transfer likely contributes substantially to the dissemination of *optrA*, although clonal expansion may also play a role in certain lineages (Figure 3). While minor variations in gene content, order, and orientation were observed in some isolates, the *poxtA*-flanking regions remained largely conserved, indicating that horizontal gene transfer plays an important role in the dissemination of *poxtA*. These findings emphasize the critical role of horizontal gene transfer and mobile genetic elements in the widespread spread of *poxtA* across genetically diverse strains.

### 3.6 Phylogenetic relationships and genomic context of *optrA*-positive isolates

This study analyzed 155 *optrA*-positive isolates using core genome SNP-based phylogenetic analysis and comparative analysis of *optrA* gene flanking regions. Due to incomplete genome assemblies, 96 isolates with intact *optrA* flanking sequences were selected for further analysis (Supplementary Figure S2). The resulting phylogenetic tree revealed significant genetic diversity, with isolates distributed across multiple clades rather than forming a monophyletic group, suggesting that horizontal gene transfer likely contributes substantially to the dissemination of *optrA*, although clonal expansion may also play a role in certain lineages. Isolates from different hosts, regions, and ecological sources were interspersed without clear clustering by epidemiological characteristics, further supporting the role of HGT over clonal expansion. The analysis of *optrA* flanking regions showed both conserved and variable genetic structures. While some isolates shared similar genetic environments, others exhibited notable differences in gene content, order, and the presence of mobile genetic elements like insertion sequences and transposase-related genes. Interestingly, phylogenetically distant isolates sometimes shared closely related *optrA* genetic environments, while closely related strains exhibited distinct *optrA*-associated structures, indicating a decoupling between host genome phylogeny and *optrA* genetic context.

**FIGURE 4:**
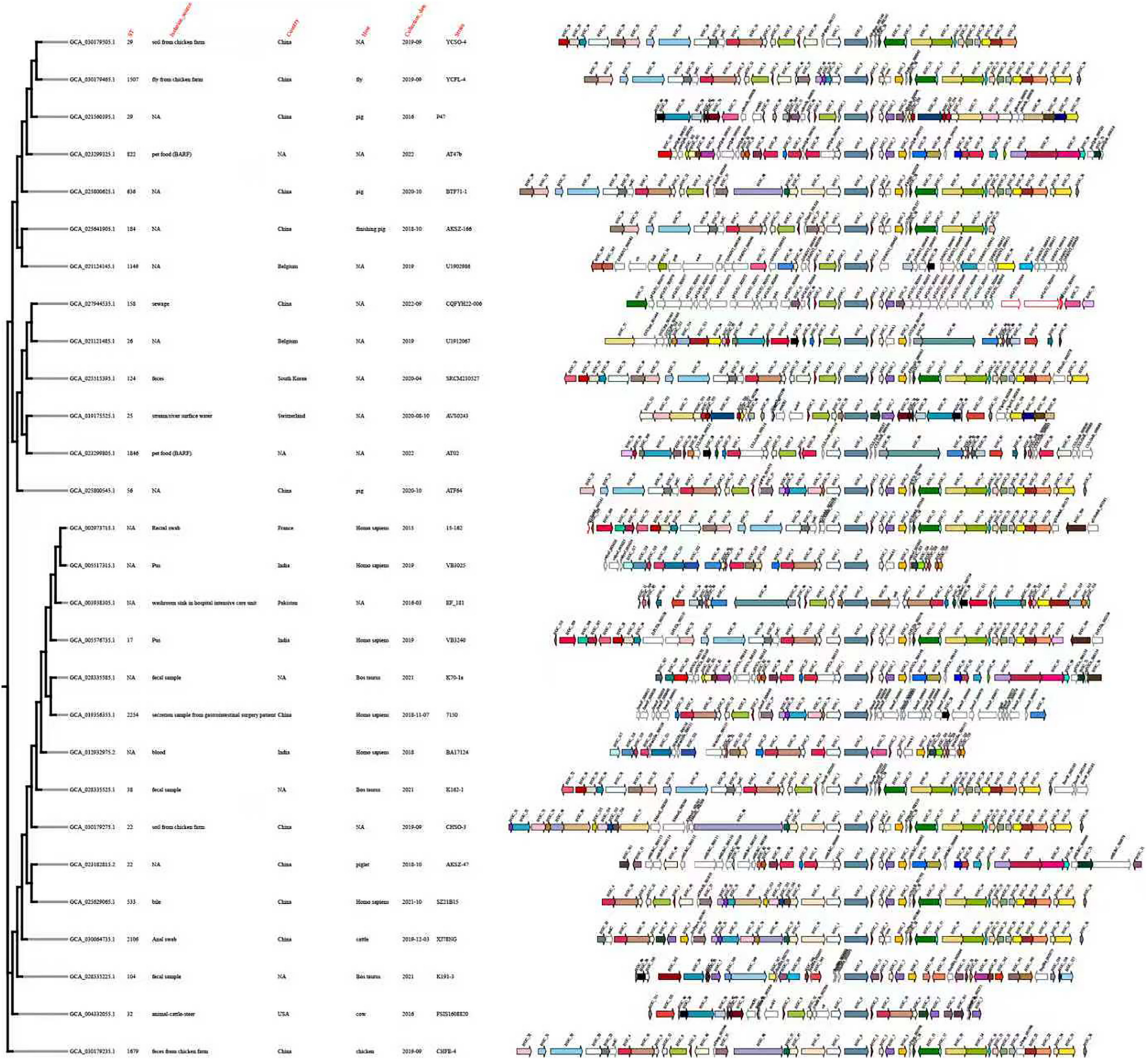
Core genome SNP-based phylogeny and diverse flanking genetic environments of 28 *optrA*-positive *Enterococcus faecium* isolates. A maximum-likelihood phylogenetic tree based on core genome single-nucleotide polymorphisms (SNPs) was constructed for 28 representative *optrA*-positive *Enterococcus faecium* isolates. These isolates were selected from a larger collection based on the presence of complete *optrA*-flanking sequences and distinct genetic structures, after excluding genetically closely related isolates with identical *optrA*-associated contexts. The phylogenetic tree is shown on the left, with corresponding metadata including isolation source, country, host, year of isolation, and strain identification. The genetic environments surrounding the *optrA* gene are displayed on the right, with arrows representing predicted open reading frames colored according to gene function. Despite the pronounced phylogenetic diversity of the host strains, the *optrA* gene is embedded within multiple distinct flanking genetic structures, many of which are associated with mobile genetic elements such as insertion sequences and transposase-related genes, highlighting the role of horizontal gene transfer in the dissemination of *optrA*.

To avoid redundancy and improve visualization clarity, representative isolates were selected based on distinct phylogenetic positions and differences in *optrA*-associated genetic environments. Isolates sharing highly similar genomic contexts and close phylogenetic relationships were grouped, and a single representative isolate from each group was retained. Using this strategy, 28 representative isolates were selected for final analysis. The maximum-likelihood phylogeny of these isolates showed pronounced genetic diversity (Figure 4), with no clear clustering based on host origin or geographic location. The flanking region analysis confirmed that the *optrA*-associated genetic environments were highly conserved across most isolates, often linked to mobile genetic elements. These findings emphasize the key role of HGT mediated by mobile elements in the widespread dissemination of *optrA* among diverse bacterial populations.

### 3.7 Phylogenetic diversity and flanking genetic contexts of *cfr*-positive isolates

**FIGURE 5:**
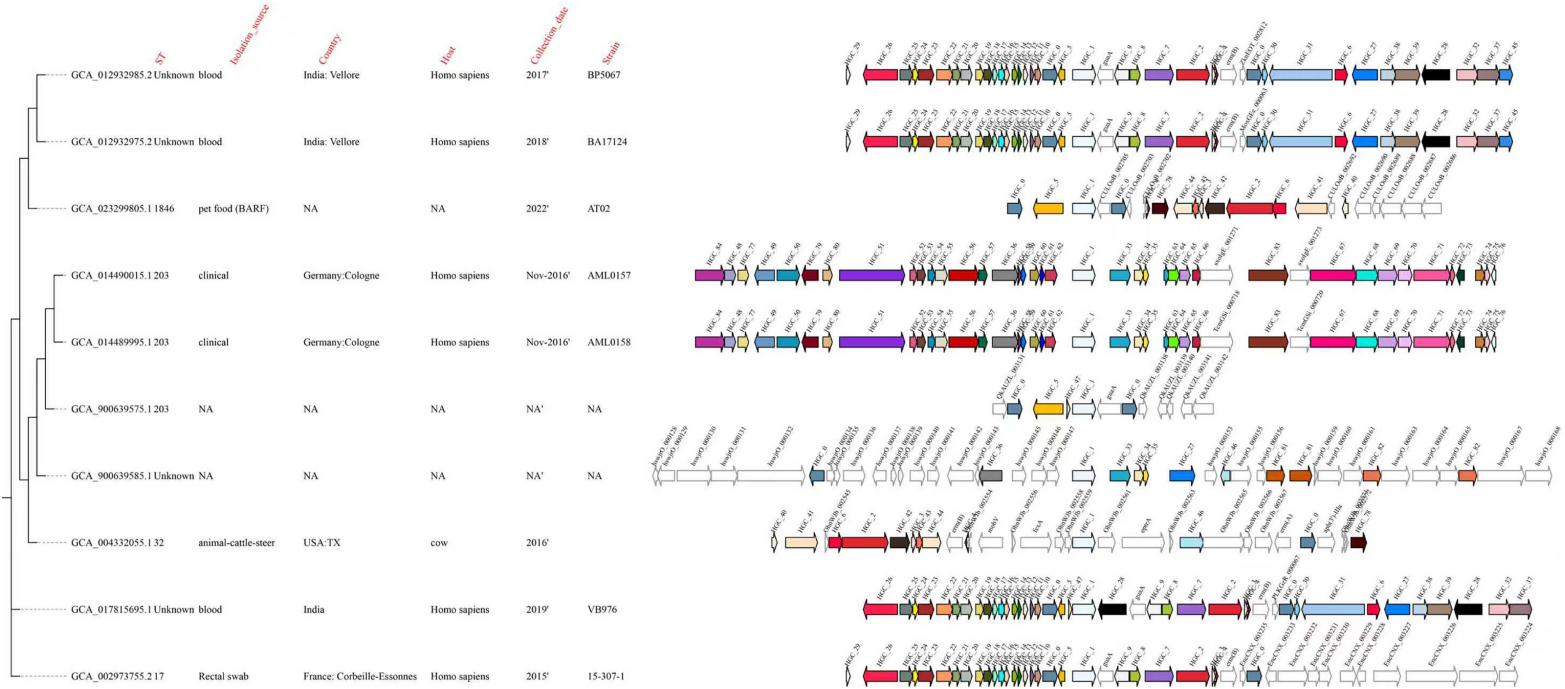
Phylogenetic relationships and flanking genetic environments of 10 *cfr*-positive isolates. A core genome single-nucleotide polymorphism (SNP)-based maximum-likelihood phylogenetic tree was constructed for 50 *cfr*-positive isolates. Due to limitations of short-read sequencing, 40 isolates exhibited substantial loss of *cfr*-flanking regions and were therefore excluded from detailed comparative analysis. Ten isolates with relatively complete genome assemblies and intact *cfr*-flanking sequences were selected for combined phylogenetic and genomic context visualization. Metadata including isolation source, geographic origin, host, collection year, and strain ID are indicated alongside the phylogenetic tree. The genetic environments surrounding the *cfr* gene are shown on the right, with arrows representing open reading frames colored according to gene function. The orientation and arrangement of genes illustrate both conserved and variable *cfr*-associated genetic structures among phylogenetically related and unrelated isolates.

Core genome SNP-based phylogenetic analysis was performed on 50 *cfr*-positive isolates. However, extensive genome fragmentation resulting from short-read sequencing led to incomplete *cfr*-flanking regions in 40 isolates, precluding detailed genetic context analysis. Due to genome fragmentation, only isolates with relatively complete genome assemblies and intact *cfr*-associated genetic regions were eligible for comparative genomic analysis. Based on these criteria, ten isolates were selected for integrated phylogenetic and genomic context analysis. The resulting phylogeny showed that these isolates were distributed across multiple distinct clades (Figure 5), indicating substantial genetic diversity and the spread of *cfr* is unlikely to be explained solely by clonal expansion. Isolates from different hosts, geographic regions, and sources were interspersed throughout the tree, with no clear epidemiological clustering, suggesting that clonal dissemination was not the primary driver of *cfr* spread. Comparative analysis of the *cfr*-flanking regions revealed both conserved and variable genetic architectures. In several isolates, *cfr* was embedded in similar gene arrangements and closely associated with mobile genetic elements, including transposases and insertion sequences, whereas distinct genetic contexts were also observed. Notably, highly similar *cfr*-associated regions were detected among phylogenetically distant isolates, while divergent contexts occasionally occurred in closely related strains, indicating a clear decoupling between host genome phylogeny and *cfr* genetic environment. Collectively, these findings support horizontal gene transfer mediated by mobile genetic elements as a key mechanism driving the dissemination of *cfr* among genetically diverse bacterial populations.

## 4 Discussion

The global distribution of transferable linezolid resistance determinants in *E. faecium* has become a growing concern because these genes can disseminate across clinical, animal, food, and environmental reservoirs (Fioriti et al., 2021; Zarzecka et al., 2022; Shen et al., 2024a). In this context, our large-scale genome mining provides a global snapshot of the presence and population diversity of *optrA*, *poxtA*, and *cfr* among publicly available *E. faecium* genomes, rather than an estimate of their true global prevalence. By integrating phylogenetic analysis with synteny comparisons, our study provides evidence consistent with an important, and likely major, role of horizontal gene transfer in shaping the spread of these determinants.

Consistent with the increasing global attention to oxazolidinone resistance in *enterococci*, transferable linezolid resistance determinants were detected in a subset of the analyzed *E. faecium* genomes, with *poxtA* and *optrA* occurring more frequently than *cfr* (Schwarz et al., 2021). This distribution pattern aligns with previous genomic and surveillance studies reporting the widespread occurrence of *poxtA* and o*ptrA* in *E. faecium* populations (Suo et al., 2025), whereas *cfr* tends to be detected less frequently in this species but remains epidemiologically important because of its broader cross-class resistance profile (Coccitto et al., 2023). Importantly, these proportions reflect frequencies within the analyzed collection of publicly available genomes and should not be interpreted as estimates of global prevalence, as genome repositories are inherently influenced by sampling biases related to geography, host source, and study objectives. Nevertheless, the presence of these determinants in a notable fraction of available genomes highlights the dissemination potential of transferable oxazolidinone resistance genes in *E. faecium*.

Another notable feature of the dataset is the occurrence of multiple linezolid resistance determinants within individual isolates. The presence of gene combinations, particularly the frequent *optrA* + *poxtA* pairing, suggests that these determinants may accumulate within the same genomic backgrounds through repeated acquisition events. This observation is consistent with previous reports indicating that oxazolidinone resistance genes are often associated with transferable plasmids and transposon-related structures (Fioriti et al., 2021; Dos Santos et al., 2026), which can facilitate their co-localization and co-transfer among *enterococci*. From an evolutionary and epidemiological perspective, the coexistence of multiple resistance genes may enhance the persistence of linezolid resistance in bacterial populations through co-selection, whereby selective pressure acting on one determinant indirectly maintains others (Pal et al., 2015). However, because most publicly available genomes are derived from short-read assemblies, the exact genomic linkage of these genes cannot always be resolved. Future studies using long-read sequencing will therefore be important for clarifying whether these resistance determinants are physically located on the same mobile genetic elements.

At the population level, a key observation from our dataset is the broad distribution of these resistance genes across multiple STs, rather than confinement to a single dominant epidemic lineage. Such a pattern is consistent with international reports showing that *optrA* and *poxtA* frequently occur in genetically diverse backgrounds in both hospital and non-hospital settings (Kuroda et al., 2018; Shen et al., 2024b), supporting the view that plasmid/transposon-mediated horizontal gene transfer plays an important role in their dissemination. At the same time, a minor contribution from clonal expansion cannot be completely excluded, particularly within specific regional or lineage-associated contexts. Previous studies have documented the occurrence of *optrA* and *poxtA* on highly similar plasmids recovered from isolates of different hosts and environments, including livestock and environmental sources (Wang et al., 2015; Hao et al., 2019; Moure et al., 2020; Biggel et al., 2021; Hou et al., 2024), highlighting the One Health dimension of these resistance determinants. Similarly, studies from China have shown that linezolid resistance gene carriers are not restricted to clinical environments, and that *poxtA*-positive *E. faecium* may circulate within broader ecological transmission networks (Wang et al., 2023).

In addition to resistance determinants, the isolates analyzed in this study harbored a diverse repertoire of VFs, with adherence-associated genes representing the largest functional group. Several virulence genes, including *eno, plr/gapA, efaA, cpsB/cdsA*, and *bopD*, were highly conserved across both resistance gene–positive and resistance gene–negative isolates, suggesting that these traits represent a stable virulence background in *E. faecium* rather than features specifically linked to linezolid resistance. At the same time, the increased prevalence of certain adherence-associated genes, such as *ecbA/fss3* and *sgrA*, in isolates carrying multiple resistance genes indicates that some genomic backgrounds may simultaneously harbor traits related to antimicrobial resistance and host colonization. Such combinations could potentially enhance the ability of these strains to persist and disseminate across clinical and non-clinical environments (Chong et al., 2025). Nevertheless, because the enrichment patterns were not uniform across all VFs, the relationship between resistance gene burden and virulence potential is likely complex and should be interpreted cautiously. Further studies integrating complete genome assemblies and phenotypic characterization will be necessary to determine whether the co-occurrence of resistance determinants and specific VFs confers measurable advantages in transmission or ecological fitness.

Our comparative analysis of genomic context further supports the contribution of mobile genetic elements to dissemination. Internationally, IS1216E has repeatedly been implicated in mobilization and cointegrate formation (Wang et al., 2023), and persistence of *poxtA*-carrying modules in *E. faecium* plasmids, and similar IS/transposase associations have been described for *optrA* and *cfr* (Shen et al., 2024b). This framework may explain a recurring pattern seen in our dataset and in previous studies: highly similar resistance-gene neighborhoods can occur in phylogenetically distant hosts, whereas closely related strains may harbor distinct resistance modules (Partridge et al., 2018). Such phylogeny-context incongruence is widely regarded as being compatible with horizontal gene transfer-driven dissemination and supports the view that conjugative and mobilizable plasmids are likely among the major vehicles involved in the spread of oxazolidinone resistance in *enterococc*i (Partridge et al., 2018). Nevertheless, these observations do not fully rule out the possibility that clonal expansion also contributes to the persistence or regional amplification of some resistant lineages.

An important strength and novel aspect of this study is the integrative genomic framework used to investigate transferable linezolid resistance determinants in *E. faecium*. Rather than focusing solely on the presence of individual resistance genes, we combined large-scale screening of publicly available genomes with analyses of phylogenetic distribution, resistance gene co-occurrence, genomic context comparisons, and virulence factor profiles. This approach allowed us to place *optrA*, *poxtA*, and *cfr* within a broader population and evolutionary context, revealing not only their distribution across diverse STs but also their frequent occurrence in multiple-gene combinations within single genomes. Furthermore, by examining the distribution of VFs among isolates carrying different numbers of linezolid resistance genes, our analysis provides additional insight into the genomic backgrounds in which these determinants occur. The enrichment of certain adherence-associated VFs in isolates harboring multiple resistance determinants suggests that some *E. faecium* lineages may simultaneously accumulate traits related to antimicrobial resistance and host colonization (Peng et al., 2022). Although these associations do not necessarily imply a direct functional linkage, they highlight the potential for resistant strains to persist and disseminate across diverse ecological reservoirs. Together, this integrative analysis provides a broader comparative perspective on the population diversity, mobilization-related contexts, and potential ecological fitness of transferable linezolid resistance genes in *E. faecium* within the currently available global genomic dataset.

Several limitations should be considered when interpreting these findings. First, our analysis relies on publicly available genomes, which may introduce heterogeneity and representation bias due to differences in sequencing capacity and surveillance infrastructure across countries. In particular, isolates from regions with limited microbiological and genomic surveillance—especially some low- and middle-income countries—may be underrepresented, potentially leading to an underestimation of the global distribution of linezolid resistance genes. Therefore, the observed gene frequencies should not be interpreted as population-level prevalence, although rigorous quality-control procedures were applied to improve data reliability. In addition, short-read genome assemblies may fragment resistance-associated regions, limiting high-confidence comparisons of flanking genetic contexts and mobile elements. Finally, although virulence-associated genes were analyzed, the regulatory mechanisms controlling resistance gene expression, including promoter regions and other regulatory elements, were not systematically assessed, and the genomic presence of resistance and virulence genes does not necessarily reflect their functional expression or clinical impact. Despite these limitations, the consistent signals observed across phylogenetic and gene-context analyses support the robustness of our inference that horizontal gene transfer plays a key role in the global dissemination of transferable linezolid resistance genes in *E. faecium*.

## 5 Conclusion

In conclusion, this study provides a large-scale genomic overview of transferable linezolid resistance determinants in *E. faecium* based on publicly available genomes. The resistance genes *poxtA*, *optrA*, and *cfr* were distributed across diverse STs and ecological sources, with *poxtA* and *optrA* detected more frequently than *cfr*. The frequent co-occurrence of resistance genes, particularly *optrA* + *poxtA*, together with phylogenetic and genomic context analyses, supports a major role of horizontal gene transfer mediated by mobile genetic elements in their dissemination. In addition, resistant isolates retained conserved virulence-associated traits, suggesting that some *E. faecium* lineages may combine antimicrobial resistance with colonization-related features. These findings provide a broader genomic framework for understanding the dissemination of linezolid resistance in *E. faecium*.

## Data Availability

The datasets used and/or analyzed during the current study are available from GenBank and the accession number and web link to datasets for the provided name of these strains are shown in Supplementary Datasheet 1.

## Footnotes

1
https://www.ncbi.nlm.nih.gov/genome/browse/#!/overview/

2
https://pubmlst.org/organisms/enterococcus-faecium

3
https://github.com/tseemann/mlst

4
https://github.com/tseemann/abricate

## Supplementary material

Supplementary Figure S1 Phylogenetic tree and *poxtA* gene flanking structures of 62 *poxtA*-positive isolates. A total of 182 *poxtA*-positive isolates were initially analyzed based on core genome single-nucleotide polymorphisms (SNPs) to construct a phylogenetic tree and examine the *poxtA* flanking regions. Due to incomplete sequencing, only 62 isolates with complete *poxtA* flanking regions were selected for further detailed analysis. The phylogenetic tree is shown on the left, illustrating the genetic diversity of these isolates, which are distributed across multiple distinct clades. Metadata for each isolate, including sample source, collection year, species, and strain information, is indicated next to the phylogenetic tree. On the right, the *poxtA* gene flanking regions are displayed for each isolate, with genes shown as arrows colored by their function. Conserved and variable genetic structures are observed in the *poxtA*-associated regions, reflecting both similarities and differences in the genetic contexts among these isolates.

Supplementary Figure S2 Core genome SNP-based phylogeny and flanking genetic environments of *optrA*-positive isolates. A total of 155 *optrA*-positive isolates were initially subjected to phylogenetic analysis based on core genome single-nucleotide polymorphisms (SNPs) and comparative analysis of *optrA* flanking genetic regions. Due to incomplete genome assemblies and fragmentation of *optrA*-associated regions, only 96 isolates with complete *optrA* flanking sequences were retained for subsequent analysis and visualization. The core genome SNP-based maximum-likelihood phylogenetic tree is shown on the left, with associated metadata including isolation source, geographic origin, host, and strain information. The genetic environments surrounding the *optrA* gene are displayed on the right, with arrows representing predicted open reading frames colored according to gene function. Conserved and variable *optrA*-associated genetic structures are observed across phylogenetically related and unrelated isolates.
