# Supplementary figures and images for: Genomic insights into the global dissemination of linezolid resistance genes in Enterococcus faecium
Source: Front Microbiol. 2026 Apr 7;17:1783172. doi: 10.3389/fmicb.2026.1783172 (PMC13095607; doi:10.3389/fmicb.2026.1783172)

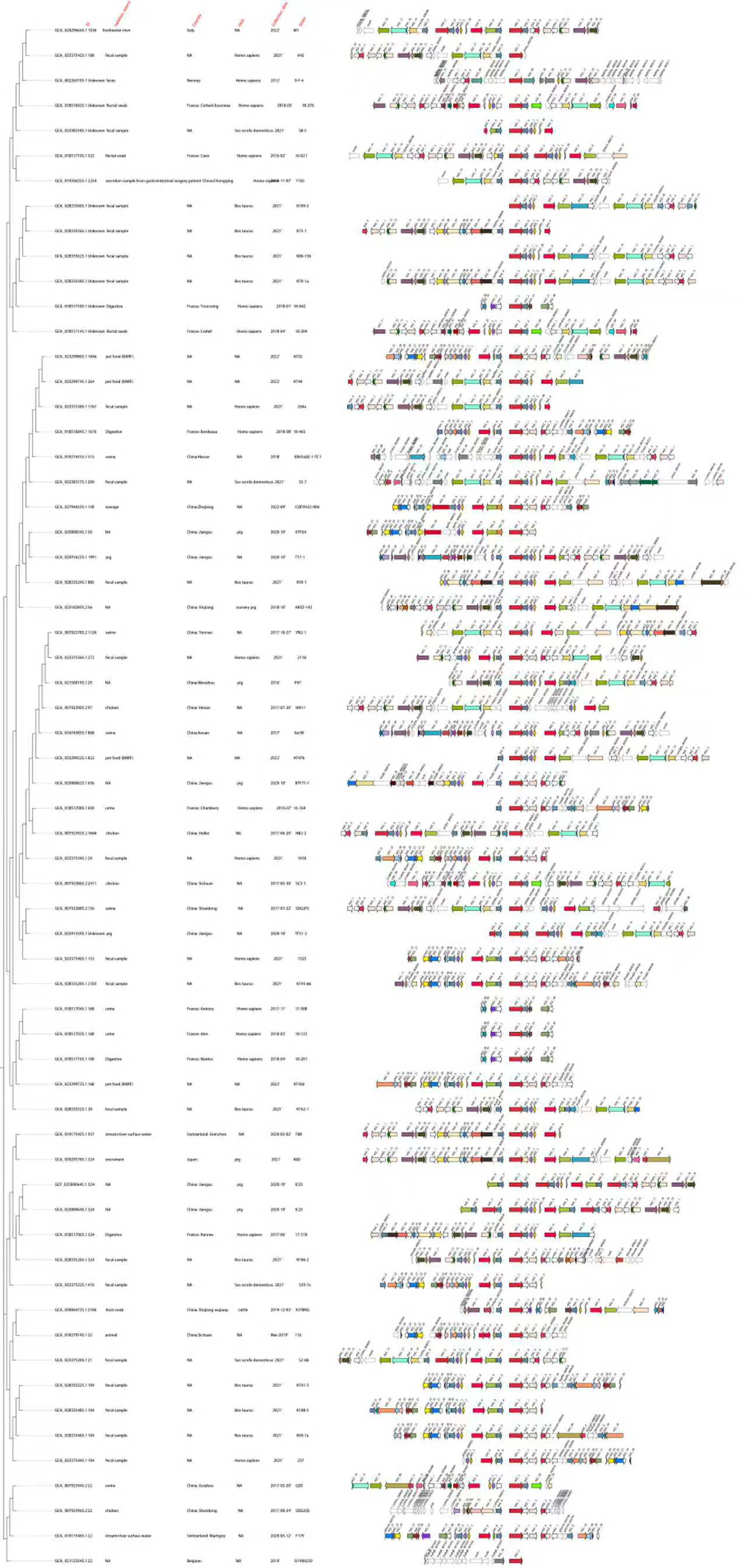

Supplement: Supplementary Figure S1 — Phylogenetic tree and poxtA gene flanking structures of 62 poxtA-positive isolates. A total of 182 poxtA-positive isolates were initially analyzed based on core genome single-nucleotide polymorphisms (SNPs) to construct a phylogenetic tree and examine the poxtA flanking regions. Due to incomplete sequencing, only 62 isolates with complete poxtA flanking regions were selected for further detailed analysis. The phylogenetic tree is shown on the left, illustrating the genetic diversity of these isolates, which are distributed across multiple distinct clades. Metadata for each isolate, including sample source, collection year, species, and strain information, is indicated next to the phylogenetic tree. On the right, the poxtA gene flanking regions are displayed for each isolate, with genes shown as arrows colored by their function. Conserved and variable genetic structures are observed in the poxtA-associated regions, reflecting both similarities and differences in the genetic contexts among these isolates. [file Image_1.JPEG]

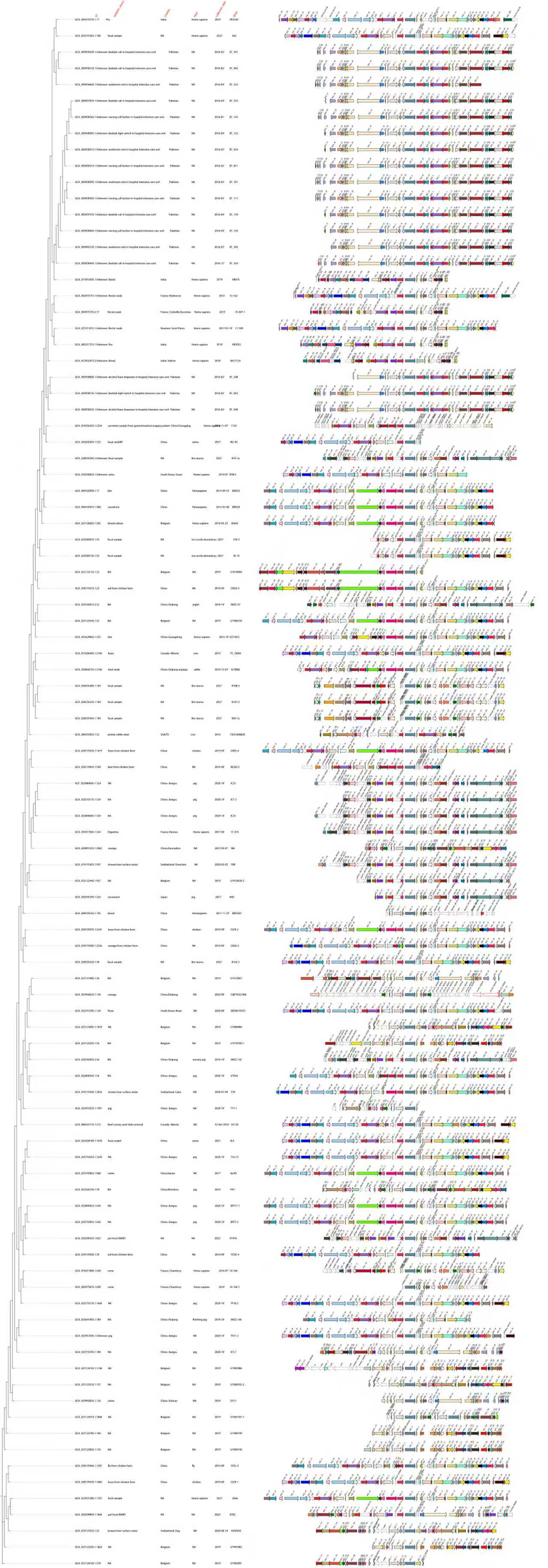

Supplement: Supplementary Figure S2 — Core genome SNP-based phylogeny and flanking genetic environments of optrA-positive isolates. A total of 155 optrA-positive isolates were initially subjected to phylogenetic analysis based on core genome single-nucleotide polymorphisms (SNPs) and comparative analysis of optrA flanking genetic regions. Due to incomplete genome assemblies and fragmentation of optrA-associated regions, only 96 isolates with complete optrA flanking sequences were retained for subsequent analysis and visualization. The core genome SNP-based maximum-likelihood phylogenetic tree is shown on the left, with associated metadata including isolation source, geographic origin, host, and strain information. The genetic environments surrounding the optrA gene are displayed on the right, with arrows representing predicted open reading frames colored according to gene function. Conserved and variable optrA-associated genetic structures are observed across phylogenetically related and unrelated isolates. [file Image_2.JPEG]
